# Effect of Wasabi Component 6-(Methylsulfinyl)hexyl Isothiocyanate and Derivatives on Human Pancreatic Cancer Cells

**DOI:** 10.1155/2014/494739

**Published:** 2014-01-20

**Authors:** Yu-Jen Chen, Yu-Chuen Huang, Tung-Hu Tsai, Hui-Fen Liao

**Affiliations:** ^1^Department of Radiation Oncology, Mackay Memorial Hospital, Taipei 104, Taiwan; ^2^Institute of Traditional Medicine, School of Medicine, National Yang Ming University, Taipei 112, Taiwan; ^3^Department of Medical Research, China Medical University Hospital, Taichung 404, Taiwan; ^4^School of Chinese Medicine, China Medical University, Taichung 404, Taiwan; ^5^Department of Education and Research, Taipei City Hospital, Taipei 103, Taiwan; ^6^Department of Biochemical Science and Technology, National Chiayi University, Chiayi 600, Taiwan

## Abstract

The naturally occurring compound 6-(methylsulfinyl)hexyl isothiocyanate (6-MITC) was isolated from *Wasabia japonica* (Wasabi), a pungent spice used in Japanese food worldwide. The synthetic derivatives 6-(methylsulfenyl)hexyl isothiocyanate (I7447) and 6-(methylsulfonyl)hexyl isothiocyanate (I7557) are small molecule compounds derived from 6-MITC. This study aimed to evaluate the effect of these compounds on human pancreatic cancer cells. Human pancreatic cancer cell lines PANC-1 and BxPC-3 were used to perform an MTT assay for cell viability and Liu's stain for morphological observation. The cell cycle was analyzed by DNA histogram. Aldehyde dehydrogenase (ALDH) activity was used as a marker for cancer stem cells (CSC). Western blotting was performed for the expression of proteins related to CSC signaling. The results showed that compounds 6-MITC and I7557, but not I7447, inhibited viability of both PANC-1 and BxPC-3 cells. Morphological observation showed mitotic arrest and apoptosis in 6-MITC- and I7557-treated cells. These two compounds induced G2/M phase arrest and hypoploid population. Percentages of ALDH-positive PANC-1 cells were markedly reduced by 6-MITC and I7557 treatment. The expression of CSC signaling molecule SOX2, but not NOTCH1, ABCG2, Sonic hedgehog, or OCT4, was inhibited by 6-MITC and I7557. In conclusion, wasabi compounds 6-MITC and I7557 may possess activity against the growth and CSC phenotypes of human pancreatic cancer cells.

## 1. Introduction

The naturally occurring compound 6-(methylsulfinyl)hexyl isothiocyanate (6-MITC) was isolated from *Wasabia japonica* (wasabi), a pungent spice used in Japanese food worldwide. This compound has been reported as having anti-inflammatory [1], chemopreventive [2], and antimelanoma [3] activities. The synthetic compounds derived from 6-MITC include 6-(methylsulfenyl)hexyl isothiocyanate (I7447) and 6-(methylsulfonyl)hexyl isothiocyanate (I7557).

Pancreatic cancer is a malignancy with increasing incidence and has been the fourth leading cause of cancer related death [4]. Due to the difficulty in making an early diagnosis, an unresectable stage at diagnosis in the majority, and resistance to chemotherapy or radiotherapy, the prognosis is poor with a 5-year survival rate of only 5–25% even after aggressive treatment [5].

It has been demonstrated that cancer stem cells (CSC) are crucial factors for treatment resistance and metastasis in many types of malignancies, including pancreatic cancer [6, 7]. Several signal transduction pathways involve the development and survival of CSC, such as Sonic hedgehog (SHH) [8], NOTCH1 [9], OCT4 [10], Wnt/beta-catenin [11], drug transporters [12], Bmi [13], and SOX2 [14]. Targeting these CSC-related signaling pathways to augment cancer control has been extensively investigated [15].

The identification of CSC is a critical issue for the evaluation of prognosis and treatment outcome, particularly in the targeting therapy. Among the biomarkers of CSC, aldehyde dehydrogenase (ALDH) has been used as one of the most common markers in investigations involving CSC, especially for the CSC of pancreatic cancer [16].

In the present study, we evaluated the effects of wasabi compound 6-MITC and its chemical derivatives on human pancreatic cancer cells, the CSC population, and signaling molecules.

## 2. Methods

### 2.1. Cell Culture

The human pancreatic cancer cell lines PANC-1 and BxPC-3 were purchased from the American Type Culture Collection (ATCC, Rockville, MD). Cells were cultured in DMEM (Biosource, Camarillo, CA) supplemented with 10% heat-inactivated fetal bovine serum (Biological Industries, Israel) at 37°C in a humidified 5% CO_2_ incubator. Cells were passaged every 2 to 3 days with a solution containing 0.25% trypsin, 0.1% EDTA, and 0.05% glucose in Hanks' balanced salt solution and maintained in exponential growth.

### 2.2. Reagents

**Figure 1:**
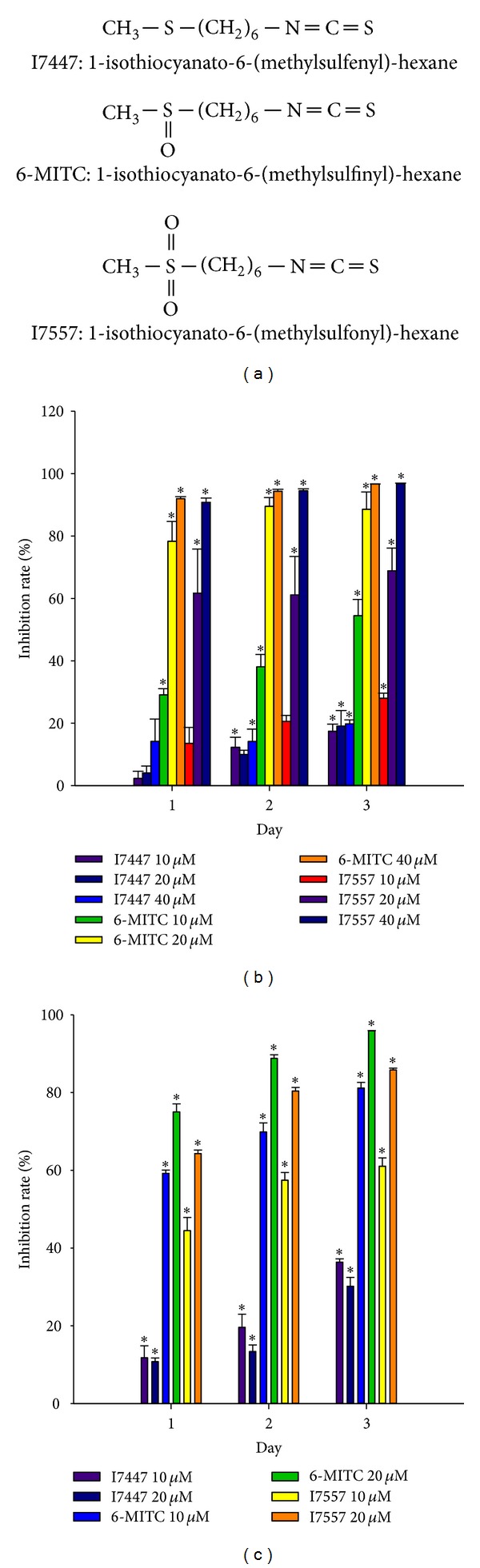
Chemical structure formulae and effects of Wasabi compound 6-MITC and derivatives on viability of pancreatic cancer cells. (a) Chemical structure formulae of I7447, 6-MITC, and I7557. (b) Viability of PANC-1 cells. (c) Viability of BxPC-3 cells. Data from three separate experiments were expressed as mean ± standard error of the mean (compared with the control group: *P < 0.05).

The compounds 6-MITC, I7447, and I7557 were purchased from LKT Laboratories (St. Paul, MN) and were dissolved in DMSO.  Figure 1(a) indicates the chemical structure formulae of I7447, 6-MITC, and I7557.

### 2.3. Cell Viability

The numbers of viable cells were estimated by using a 3-(4,5-dimethylthiazol-2-yl)-2,5-diphenyltetrazolium bromide (MTT, Sigma) colorimetric assay.

### 2.4. Morphology

The cells were stained by Liu's stain. Liu A solution was added for 45 seconds at room temperature, followed by adding Liu B solution for 90 seconds. Then cells were gently washed and the cell morphology was observed by light microscope (Olympus, Tokyo, Japan) at a magnification of 1000. Photographs were taken with a digital camera (Olympus, Tokyo, Japan).

### 2.5. Aldehyde Dehydrogenase Activity Assay

Aldehyde dehydrogenase (ALDH) activity, a hallmark of CSC [16], was measured by using an ALDEFLUOR assay system kit (STEMCELL technologies Inc., Taipei, Taiwan).

### 2.6. Western Blotting

Whole-cell lysates were prepared from cells with various treatments. The polyvinylidene fluoride membrane was blocked with 5% defatted milk and then immunoblotted with primary antibodies, including NOTCH1, SHH, ABCG2, OCT4, SOX2, and beta-actin (BD Transduction Laboratories), at room temperature for 2 hours. This was followed by adding horseradish peroxidase-labeled second antibodies (Chemicon, Single Oak Drive Temecula, CA) and was developed using the enhanced chemiluminescence system (Amersham Pharmacia, Piscataway, NJ). The expression of beta-actin was used as an internal control.

### 2.7. Statistical Analysis

Data are presented as means ± standard error of the mean (SEM). One-way analysis of variance followed by Dunnett's test was used to compare the inhibition rate among different treatments. Statistical analyses were performed using the SPSS software package (version 18.0) and *P* value less than 0.05 was considered significant.

## 3. Results

### 3.1. Effect of Wasabi Compounds on Viability of Pancreatic Cancer Cells

The natural Wasabi compound 6-MITC and its chemical derivative I7557, but not I7447, inhibited viability of both PANC-1 and BxPC-3 cells in a dose- and time-dependent manner (Figures 1(b) and 1(c)).

### 3.2. Morphology

**Figure 2:**
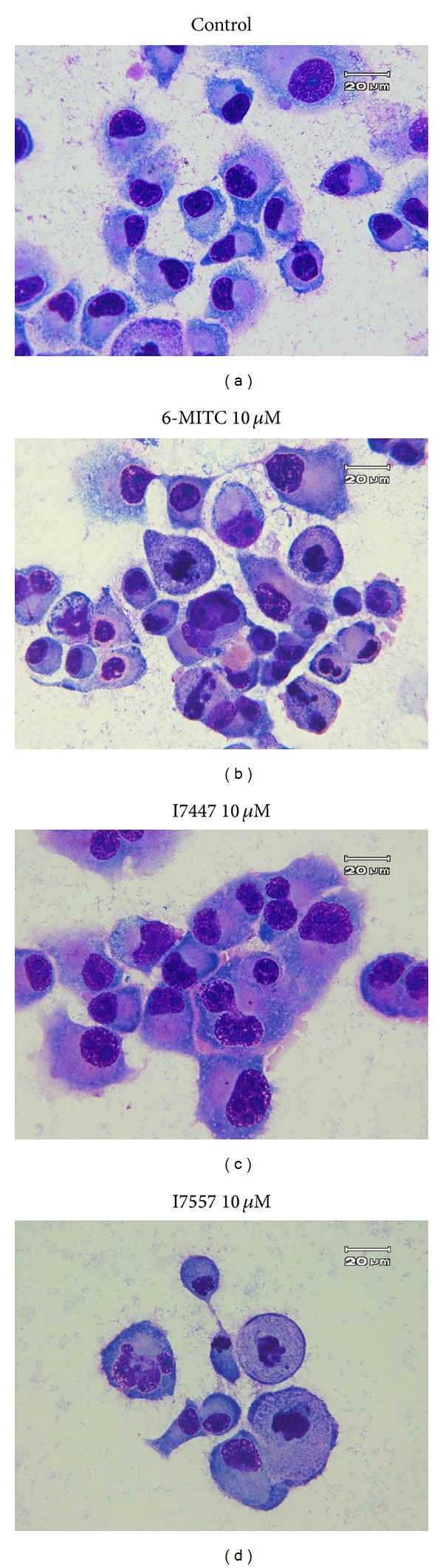
Morphology of PANC-1 cells treated by Wasabi compound 6-MITC and derivatives. Cells were treated with 6-MITC, I7447, and I7557 at 10 *μ*M for 24 hours. Magnification 1000x.

As demonstrated in Figure 2, morphological observation showed marked features of mitotic arrest and apoptosis in 6-MITC- and I7557-treated cells for 24 hours.

### 3.3. Cell Cycle Analysis

**Figure 3:**
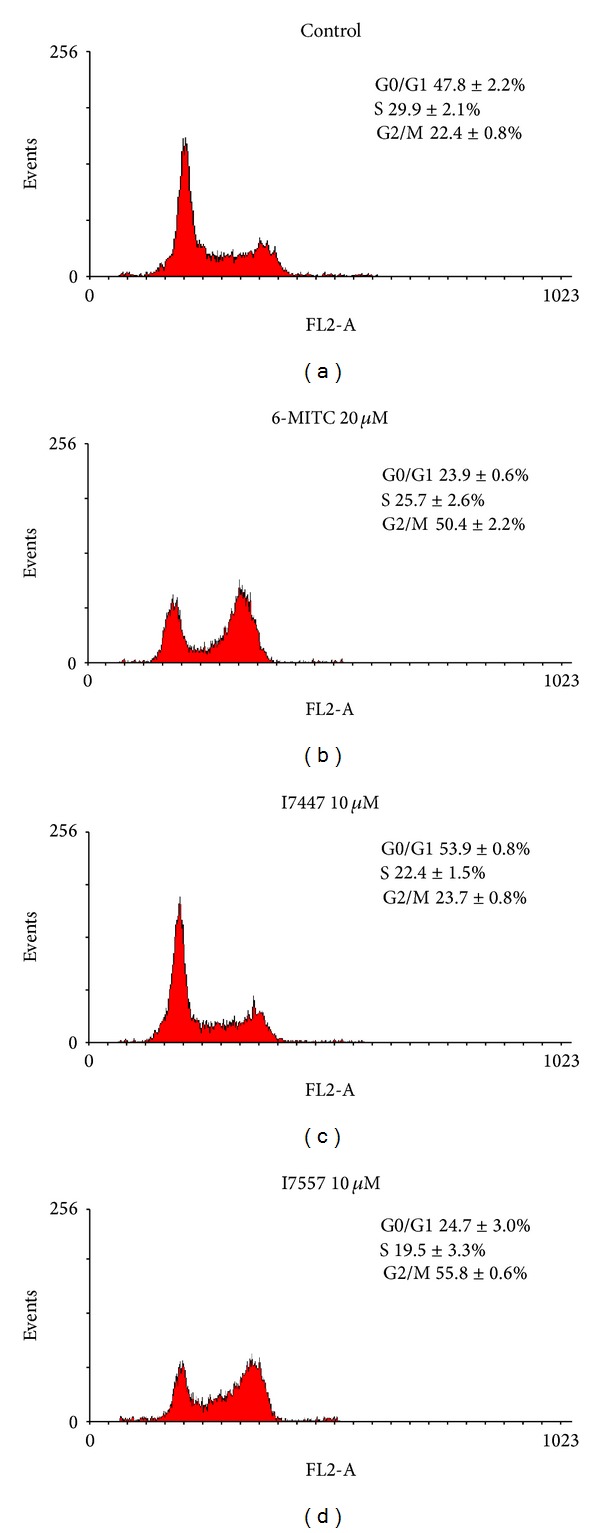
Cell cycle analysis of PANC-1 cells treated by Wasabi compound 6-MITC and derivatives. Cells were treated with 6-MITC, I7447, and I7557 at 20 *μ*M for 24 hours. Data from 3 separate experiments were expressed as mean ± standard error of the mean.

Cell cycle analysis revealed an increase in percentage of G2/M phase in PANC-1 cells from 22.4 ± 0.8% of controls to 50.4 ± 2.2% of 6-MITC-treated group and 55.8 ± 0.6% of I7557-treated group at 20 *μ*M (Figure 3). The percentages of apoptotic cells estimated by hypoploid population were increased by 6-MITC and I7557 as well.

### 3.4. Alteration in CSC Population

**Figure 4:**
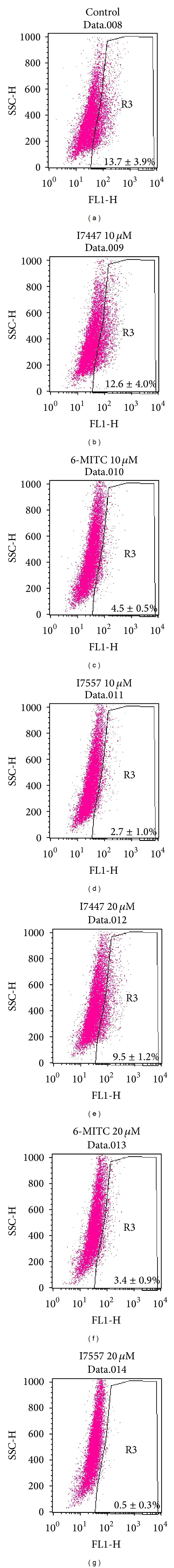
Effect of Wasabi compound 6-MITC and derivative on ALDH activity of PANC-1 cells. Cells were treated with 6-MITC, I7447, and I7557 for 24 hours. Data from 3 separate experiments were expressed as mean ± standard error of the mean.

Percentages of ALDH-positive PANC-1 cells, as a marker of CSC, were markedly reduced by 10 *μ*M 6-MITC and I7557 treatments from 13.7 ± 3.9% in the control group to 4.5 ± 0.5% in the 6-MITC-treated group and 2.7 ± 1.0% in the I7557-treated group. This ratio was further reduced in the 6-MITC-treated group to 3.4 ± 0.9% and to 0.5 ± 0.3% in the I7557-treated group by incremental increases in the concentration of 6-MITC and I7557 to 20 *μ*M (Figure 4).

### 3.5. Expression of CSC Signaling Proteins

**Figure 5:**
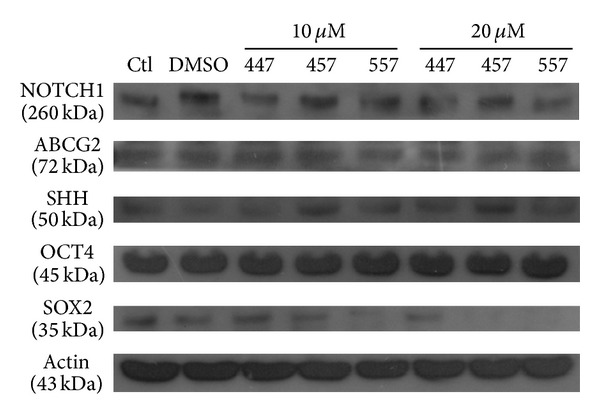
Expression of CSC-related signaling molecules in PANC-1 cells. Cells were treated with 10–20 *μ*M of 6-MITC, I7447, and I7557 for 24 hours and then subjected to Western blotting.

By treatment with 6-MITC and I7557, the expression of SOX2, but not NOTCH1, ABCG2, Sonic hedgehog, or OCT4, was inhibited in PANC-1 cells (Figure 5).

## 4. Discussion

The naturally occurring bioactive compound 6-MITC from wasabi and its chemical derivative I7557 were demonstrated to have bioactivity against human pancreatic cancer cells in this study. Furthermore, the CSC population and the expression of SOX2 were reduced by treatment with these compounds.

As a pungent spice used in food, especially in Japanese cuisine, wasabi is commonly accepted for use in a small amount. Due to the strong flavor, the use of wasabi as a food condiment may not be suitable for treatment or prevention of cancer. Instead, the pure compound 6-MITC and its chemical derivative I7557, which are flavorless, might be more suitable for development as bioactive agents against cancer.

To overcome CSC characteristics, small molecules targeting CSC signaling pathways are promising and under way in clinical trials. For example, GDC-0449 could reduce the tumor burden of basal-cell carcinoma and block the growth of new tumors in patients with basal-cell nevus syndrome. However, adverse effects caused the discontinuation of treatment in over half of treated patients [17]. This implies that it remains necessary to develop novel agents targeting CSC signaling pathways that do not have major adverse effects.

SOX2 (SRY-related HMG-box gene 2) was initially reported to be linked with the inhibition of neuronal differentiation due to its action as a transcriptional factor in the maintenance of the self-renewal capability of embryonal stem cells [18]. It has been demonstrated that it is a novel target of EGFR-Src-Akt signaling in non-small-cell lung cancer, modulating self-renewal and the expansion of cancer stem-like cells [19]. The downregulation of SOX2 by 6-MITC and I7557 in PANC-1 pancreatic cancer cells harboring mutant K-ras may shed light on elucidating the interaction between SOX2, EGFR, and its downstream KRAS/BRAF and PI3K/AKT pathways. Given that more than 90% of clinical specimens of pancreatic duct adenocarcinoma possess mutant K-ras, the effect of SOX2 downregulation by 6-MITC and I7557 in PANC-1 cells may have potential for further preclinical investigation. Whether the downregulation of SOX2 by 6-MITC and I7557 in PANC-1 cells is associated with the signaling of KRAS/BRAF, PI3K/AKT, and EGFR pathways remains to be elucidated.

In conclusion, Wasabi compound 6-MITC and its chemical derivative I7557 may possess bioactivity against human pancreatic cancer cells, including the CSC population.
